# Prevalence of foot disorders in lactating Jersey cows raised in semi-confinement

**DOI:** 10.14202/vetworld.2020.2612-2617

**Published:** 2020-12-09

**Authors:** B. F. Matias, L. F. C. Cunha Filho, F. C. A. Rego, J. V. P. Barreto, L. S. L. S. Reis, A. T. Z. Queiroz, G. R. Queiroz

**Affiliations:** 1 Master´s Degree in Animal Health and Production, Pitágoras Unopar University, Arapongas, PR, Brazil; 2 University of Western Paulista, Sao Paulo, Brazil

**Keywords:** dairy cow, digital disease, lameness

## Abstract

**Background and Aim:**

To establish prevention strategies, recording the prevalence of foot injuries within a herd should be the starting point in determining the risk factors involved in digital diseases. This study aimed to assess the prevalence of claw disorders in lactating Jersey cows raised in a semi-confinement system.

**Materials and Methods:**

Five hundred and eighty-four digits were examined from 73 lactating Jersey cows. The lameness score system was used to assess each cow while walking and on standing position, and digital lesions were evaluated with the cows restrained in a hydraulic cattle chute.

**Results:**

The prevalence of digital lesions was 93.1%. Among the 68 affected cows, only 21 were lame. Of the 584 digits examined, 970 lesions were recorded, corresponding to 13.3% lesions per cow and 1.66% injuries per digit. Forty-eight cows (65.7%) had lesions in all digits, and 92.8% of digits had at least one lesion. Lesions in digits of fore limbs were more common (55.3%) (p<0.0001) than those of hind limbs (44.7%). Foot injuries in medial digits of the fore limbs were more prevalent (56.8%) (p<0.001) than in the lateral digits (43.2%). The lesions’ occurrence was similar in both medial and lateral digits of the hind limbs (p=0.8347). The primary diseases observed were heel horn erosion (53.8%), white line disease (19.3%), and double sole (12.4%), which together accounted for 92.4% and 84.9% of foot disorders diagnosed in the fore and hind limbs, respectively. Other digital diseases occurred less frequently.

**Conclusion:**

The prevalence of foot disorders in lactating Jersey cows raised in semi-confinement systems was high. This could be due to the lack of preventive trimming, infectious diseases, and nutritional problems.

## Introduction

Brazilian dairy cattle rearing has improved a lot in the last decades, mainly about cows’ productivity and the use of technology involved in the production system. Semi-confinement systems have become an economically viable practice in milk production in the country [1], unlike the tie-stall and free-stall systems, in which cows remain in the shed all the time. The semi-confinement system has proven to be more profitable and should be implemented with the comfort and well-being of animals in mind, so productivity is not compromised [2]. However, the control of digital injuries can be a challenge since the environmental factors in this system can increase these injuries.

Digital diseases are the main cause of pain and discomfort in dairy cattle, causing lameness leading to productive and reproductive losses [2,3], increased cost of treatment [4], and early disposal of animals [3,5]. In cattle, lameness is an essential concern in animal welfare [2], with 90% being attributed to hoof problems [5]. All lameness-related problems can be prevented with proper management and preventive hoof treatment [6].

Claw disorders in cattle can be classified according to their etiology and infectious and non-infectious nature [7]. Their prevalence may vary according to the production system [8,9]. In several dairy farm systems, preventive hoof trimming is practiced annually or even twice a year for the unquestionable health benefits it brings on the hooves and, consequently, on the cows [10]. To establish prevention strategies, recording the prevalence of foot injuries within a herd should be the starting point in determining the risk factors involved in digital diseases [11].

This study aimed to assess the prevalence of foot lesions in lactating Jersey cows in a semi-confinement system.

## Materials and Methods

### Ethical approval

The experiment was carried out in accordance with the guidelines of the Ethics Committee on the usage of animals in experiments and was approved by the Scientific Committee (CEA/UNOPAR 028/19).

### Animals and traits

To observe the primary claw disorders, all lactating cows’ digits, and not only of lame cows, were studied. A survey was carried out in February 2019, on a dairy farm in the northern region of the state of Paraná, Brazil, where 584 digits and adjacent regions of the hooves of 73 lactating Jersey cows, aged between 2 and 10 years old and with an average milk yield of 20 L/day, were evaluated. The cows were kept in a semi-confined regime where they remained in a containment area for approximately 12 h between the two milking daily shifts. Their diet consisted of corn silage, concentrated feed (7 kg/cow/day), and mineral salt for lactating cows (*ad libitum*). Soon after the end of the second milking shift, the cows were kept in Tifton paddocks (*Cynodon dactylon*) until the next milking. Water was provided *ad libitum* in artificial drinkers in both areas. The containment area consisted of a covered section with concrete floors and an uncovered section with a dirt floor. No bedding was provided for the cows to lie down. The mechanical milking parlor remained next to the containment shed.

### Locomotion score

A veterinarian performed the evaluation of the locomotion score among cows in season, the evaluation was based on the observation of the spinal postures of cows while standing and walking, was performed on a concrete surface, and the score range from 1 to 5 [12] was used, with score 2 being considered as the onset of claudication, indicating mildly lame (i.e., the cow that stands with a flat back but develops an arched back while walking, and has a normal gait during walking); score 3 indicates moderately lame cow (i.e., an arched-back posture is noted when the cow is in standing position and while walking, her gait is short in one or more limbs); score 4 indicates lame cow (i.e., it is easy to notice the arched-back posture during the standing position and while walking, and the gait always favors one or more limbs); and the score 5 indicates the severely lame cow (i.e., the cow still has arched back while standing and walking, but refuses or is extreme reluctant to bear weight on the limb or feet) [12].

The animals were restrained in a hydraulic cattle chute. Their digits and interdigital spaces were cleaned with soap and water. A thin layer of the hoof’s sole was removed to facilitate the examination and visualization of lesions in the sole, the bead, and the white line zones. In some specific cases, the examination was carried out using hoof forceps [13]. After examining each digit, the claw disorders were classified according to Greenough and Weaver [7] and Greenough [14], in the following classification: Heel horn erosion, sole ulcer, toe ulcer, heel ulcer, sole hemorrhage, white line disease, double sole, subsolar abscess, interdigital dermatitis, interdigital phlegmon, interdigital hyperplasia, slipper foot, corkscrew claw, digital dermatitis, and diffuse septic laminitis. In this property, preventive animal hoof trimming was not carried out, and interventions only occurred when the cows presented lameness with a score of 4/5 or 5/5 [14]. The only way foot injuries were prevented using footbaths of 5% copper sulfate solution once a month. No foot washer was provided, and the solution was not regularly changed.

### Statistical analysis

The observed information was grouped in tables according to the frequency of occurrence of foot injuries and statistically analyzed using the Chi-square test. Digital lesions were compared between the lateral and medial digits of the fore limbs and those of the hind limbs. The number of foot injuries between fore and hind limbs was also compared. The number of interdigital lesions of the fore limbs was also evaluated in comparison with the hind limbs at 5% level of significance using the software R Development Core Team (R Foundation for Statistical Computing, Vienna, Austria) [15].

## Results

Of the 73 cows examined, 68 had at least one digital lesion, representing 93.1% prevalence. However, only 28.7% of the cows (21/73) showed changes in locomotion patterns, which was classified according to scores 2/5 (5.5%, i.e., 4/73), 3/5 (5.5%, i.e., 4/73), 4/5 (12.3%, i.e., 9/73), and 5/5 (5.5%, i.e., 4/73). In the 584 digits examined, 970 injuries were observed, which corresponded, on average, to 13.3 injuries per cow and 1.7 injuries per digit.

Forty-eight cows (65.7%) had lesions in all digits, and in 92.8% of digits, at least one lesion was observed. Interestingly, the fore limb digits (55.3%) contained more injuries than the hind ones (44.7%) (p<0.0001). The sole ulcer was more frequently observed in the hind limbs, and the toe ulcer was less frequent and observed only in the hind limbs’ medial digits.

The medial digits of the fore limbs had more claw disorders (299/526; 56.8%) (p<0.001) than the lateral ones (227/526; 43.2%), and in the fore limbs, the distribution of lesions was practically equivalent, as the medial digits contained 49.4% (200/405) of the lesions and the lateral digits had 50.6% (205/405) (p=0.8347).

The three main diseases observed were heel horn erosion, white line disease, and double sole (Table-1), which together accounted for 92.4% (486/526) and 84.9% (344/405) of the foot disorders diagnosed in the fore and hind digits, respectively. Heel horn erosion represented 53.8% (523/970) of all diagnosed digital diseases and occurred more frequently in the fore limbs (53.9%). White line disease (186/970; 19.3%) also occurred more often in the fore limbs (63.4%) and was diagnosed mainly in the medial digits (74/186; 39.8%). Double sole alterations were diagnosed 121 times, with 71.0% occurring in the fore limbs. Sole hemorrhage (25/970; 2.6%) occurred more frequently in the lateral digits of the hind limbs (44%) and the medial digits of the fore limbs (36%). Lesions classified as sole ulcers occurred in 17 digits (1.7%), 7 of which were recorded in the medial digits of the fore limbs and 10 distributed equally in the medial and lateral digits of the hind limbs.

**Table-1:** Distribution and frequency of claw disorders (n [%]) by members and digits of lactating Jersey cows maintained in a semi-confined system.

Classification	LFL			RFL			LHL			RHL			Total lesions
			
	IS	LD	MD	IS	LD	MD	IS	LD	MD	IS	LD	MD	
Heel horn erosion	-	69 (7.1)	72 (7.4)	-	71 (7.3)	70 (7.2)	-	58 (5.9)	56 (5.7)	-	67 (6.9)	60 (6.0)	523 (53.9)
White line disease	-	20 (2.0)	46 (4.7)	-	24 (2.4)	28 (2.8)	-	11 (1.1)	23 (2.3)	-	20 (2.0)	14 (1.4)	186 (19.1)
Double sole	-	17 (1.7)	33 (3.4)	-	17 (1.7)	19 (1.9)	-	1 (0.1)	18 (1.8)	-	10 (1.0)	6 (0.6)	121 (12.4)
Sole hemorrhage	-	2 (0.2)	6 (0.6)	-	1 (0.1)	3 (0.3)	-	6 (0.6)	-	-	5 (0.5)	2 (0.2)	25 (2.5)
Interdigital dermatitis	2 (0.2)	-	-	-	-	-	15 (1.5)	-	-	6 (0.6)	-	-	23 (2.3)
Sole ulcer	-	-	4 (0.4)	-	-	3 (0.3)	-	1 (0.1)	2 (0.2)	-	4 (0.4)	3 (0.3)	17 (1.7)
Subsolar abscess	-	3 (0.3)	3 (0.3)	-	1 (0.1)	2 (0.2)	-	-	3 (0.3)	-	2 (0.2)	1 (0.1)	15 (1.5)
Interdigital hyperplasia	4 (0.4)	-	-	4 (0.4)	-	-	-	-	-	6 (0.6)	-	-	14 (1.4)
Slipper foot	-	-	4 (0.4)	-	-	2 (0.2)	-	2 (0.2)	-	-	2 (0.2)	2 (0.2)	12 (0.8)
Corkscrew claw	-	-	-	-	2 (0.2)	2 (0.2)	-	2 (0.2)	2 (0.2)	-	2 (0.2)	1 (0.1)	11 (1.1)
Distal phalanx osteitis	-	-	-	-	-	-	-	2 (0.2)	-	-	3 (0.3)	2 (0.2)	7 (0.7)
Claw overgrown	-	-	2 (0.2)	-	-	-	-	-	-	-	1 (0.1)	2 (0.2)	5 (0.5)
Digital dermatitis	-	-	-	-	-	-	-	2 (0.2)	-	-	1 (0.1)	-	3 (0.3)
Diffuse septic laminitis	-	-	-	-	-	-	-	2 (0.2)	1 (0.1)	-	-	-	3 (0.3)
Toe ulcer	-	-	-	-	-	-	-	-	2 (0.2)	-	-	-	2 (0.2)
Interdigital phlegmon	-	-	-	-	-	-	-	-	-	2 (0.2)	-	-	2 (0.2)
Heel ulcer										1 (0.1)		1 (0.1)
Total	6 (0.6)	111 (11.4)	170 (17.5)	4 (0.4)	116 (11.9)	129 (13.3)	15 (1.5)	87 (8.9)	107 (11.0)	14 (1.4)	118 (12.1)	93 (9.5)	970 (100)
	536 A (55.2)						434 B(44.8)						

A,B Different letters indicate difference between the amount of digital disorders (p<0.05).

LFL=Left fore limb, RFL=Right fore limb, LHL=Left hind limb, RHL=Right hind limb, IS=Interdigital space, LD=Lateral digit, MD=Medial digit

The diseases that affected the interdigital space did not occur very often, with a total of 39 lesions (4.0%), and were greater in the hind limbs (p=0.0032) compared to the fore limbs. The main interdigital lesions classified in this study were interdigital dermatitis (23/970; 2.4%) and interdigital hyperplasia (14/970; 1.4%). Interdigital dermatitis was most frequently seen in the hind limbs, representing 91.3% of cases.

In this study, the association between heel erosion and interdigital dermatitis was not observed since interdigital hyperplasia had an equivalent distribution of 57.1% and 42.9% occurring in the fore and hind limbs, respectively, and totalizing 14 injuries (1.4%). Interdigital phlegmon was diagnosed on only two occasions and always occurred in the hind limbs. Other injuries were not observed very often and represented only 6.2% of the digital diseases found (Table-1).

## Discussion

In confinement systems, 100% of cows examined had foot lesions [16], a fact similar to the present study results. Despite this, other studies performed in Brazil showed a prevalence of foot injuries of 30.2% in a confinement system [17], 40% in a semi-intensive system [4], and lower frequencies of 16.6% [4] and 22.2% [18], respectively, in an extensive milk production system. The high frequency of claw disorders recorded in the present study must be associated with the fact that all digits of all lactating cows were examined, unlike other studies, which evaluated only the digits of cows that had altered locomotion patterns [18]. Nevertheless, it is essential to note that cows that do not limp may have digital lesions [19].

Another essential factor for the high prevalence of foot disorders is that the studied property did not have an effective program for preventing foot injuries, including a periodic hoof trimming schedule followed by footbath and constant solution change.

The lameness score can be used to quickly establish the health status of the digits of a given herd on a farm [13]. In this property, the number of lame cows was very similar to that observed in semi-confinement systems (15.3%) [8] and in free-stall, where 13.7% [8] and 22.3% [19] of cows were lame. However, in another study, a high frequency of lameness was found in Holstein cows raised in the free-stall system, reaching 70.5% in multiparous cows and 32.3% in primiparous cows [16]. In the Castro dairy basin (state of Paraná, Brazil), studies were carried out on 50 properties that used production systems as compost barn, free-stall, and the association between these two systems. An average prevalence of lameness of 42.5% was observed, and among the three production system, the association of free-stall with compost barn presented the highest prevalence of cows being lame [3]. Recently, a study involving Jersey cows found the lameness prevalence of 13.6% and 15.2% in free-stall and semi-confinement systems, respectively [8]. Surprisingly, all cows with lameness were stratified between Grades 2 and 3, and there were no animals with more severe degrees of lameness [8]. On the other hand, in the actual study, 61.9% (13/21) of cows with altered locomotion patterns were classified between the scores 4 and 5, that is, the most severe degrees of lameness. This stratification in the classification of lameness tends to be different between studied properties, as it depends on several factors such as production systems and the use of preventive hoof trimming [3,8].

As for the average of claw disorders, the result of this present study agreed with other authors who reported values of 19.7 and 18.1 in primipara and multipara of the Dutch breed, respectively [16]. In Jersey cows, the average number of lesions per cow was 15.6 [8], which was very similar to this study’s result. Contradicting the result of the present study, 1225 Girolando cows were evaluated, where it was observed that there were only 64-foot lesions in 25 cows. This variability in results can be explained by the differences in production systems, in the types of preventive management applied to each property, in the genetic factors, and in the risk factors for each foot disease [7,14].

Regarding the fore and hind limbs, the present study results agreed with the results obtained who found a greater number of foot disorders on the fore limbs than the hind limbs, that is, 54.43% and 45.57%, respectively [20]. However, that study contradicted other surveys of digital lesions in cattle that observed a higher prevalence of lesions in the hind limbs, 58.9% [8], 82.1% [4], 57.26% [1], 66.6% [17], 75.3% [21], and 61.8% [18]. Corneal wear disorders, such as sole hemorrhage, sole ulcer, and toe ulcer, are responsible for 23-60% of cases of foot injuries in dairy herds in the United States [22]. In this study, the group of lesions in the corneal wear injuries represented only 4.4% of the lesions. In a survey carried out in 156 dairy farms, in which 28,607 cows were evaluated, even though the prevalence of claw disorders was higher in the hind limbs, lesions such as sole hemorrhage, sole ulcer, and toe ulcer occurred more frequently in the digits of the fore limbs, compared to diseases of infectious origin, such as digital dermatitis [5]. In the present study, the corneal wear disorders, such as sole hemorrhage, sole ulcer, and toe ulcer, were often observed in fore and hind limbs, but if the double sole disorder is added to this list, as it should because it is a laminitis-related claw disorder, it becomes more prevalent in the fore limbs.

Heel horn erosion lesions are often seen in cows kept confined [19] but have also been found in high prevalence among cows kept in other types of systems such as semi-confinement [8]. The determining factors for the occurrence of lesions are related to cattle rearing conditions, including high temperature, poor hygiene, and wet environment caused by the accumulation of feces and organic matter. The latter facilitates the contamination of the corneal bulbar tissue by *Dichelobacter nodosus*, one of the etiological agents of interdigital dermatitis [10,14]. And still, interdigital dermatitis was observed in low frequency, compared to the most prevalent lesion, heel horn erosion. The white line disease is a prevalent claw disorder in dairy cows [23] and was not different in this research; it was the second-highest disorders observed in this herd and was found in many different degrees. According to Barker *et al*. [24], the risk factors for white line disease allowed cows at pasture during the day, housed at night, concrete floors in yards or alleys, and parity. These risk factors were seen in the property studied, and the only difference was between the grazing time, that was at night, and they were housed by day.

In this study, cows remained confined all day, and the site was not cleaned daily. There are reports that cows with heel horn erosion can improve spontaneously if they are kept exclusively at pasture [10]. However, this was not the situation on this property. Even though the cows had access to pasture at night, the duration was too short and did not help control heel horn erosion. The association between heel horn erosion and interdigital dermatitis was not observed in this work, as reported by other authors in dairy cows confined in tie-stall and free-stall [25]. The semi-confinement system, where animals spend the night at pasture, may contribute to the lower frequency of interdigital dermatitis [14].

This study showed that interdigital hyperplasia had a balanced distribution with 57.1% and 42.9% occurring in the fore and hind limbs, respectively, and totaled 14 injuries (1.4%). Although interdigital hyperplasia is frequently observed in cattle raised in an extensive production system [18], its prevalence is low in more intensive milk production systems [8].

## Conclusion

Jersey lactating cows in a semi-confinement system showed a high prevalence of claw disorders, the main ones being heel horn erosion, white line disease, and double sole. The lack of preventive hoof trimming was one of the determining factors for the occurrence of these disorders. It is noted that the most prevalent disorders are known as the laminitis-related claw disorders, and the prevention of this should be toward nutrition and periodic hoof trimming.

## Authors’ Contributions

GRQ and LFCCF supervised, designed, and coordinated the study. JVPB, BFM, and LSLSR performed the experiments and collected the data. FCAR analyzed the data. GRQ and ATZQ wrote the manuscript. All authors read and approved the final manuscript.
